# Enhanced Catalytic Reduction of 4-Nitrophenol Driven by Fe_3_O_4_-Au Magnetic Nanocomposite Interface Engineering: From Facile Preparation to Recyclable Application

**DOI:** 10.3390/nano8050353

**Published:** 2018-05-22

**Authors:** Yue Chen, Yuanyuan Zhang, Qiangwei Kou, Yang Liu, Donglai Han, Dandan Wang, Yantao Sun, Yongjun Zhang, Yaxin Wang, Ziyang Lu, Lei Chen, Jinghai Yang, Scott Guozhong Xing

**Affiliations:** 1 College of Physics, Jilin Normal University, Siping 136000, China; 17649973053@163.com (Y.C.); 13944139606@163.com (Y.Z.); 13944949603@163.com (Q.K.); syt@jlnu.edu.cn (Y.S.); yjzhang@jlnu.edu.cn (Y.Z.); wangyaxin1010@126.com (Y.W.); chenlei@jlnu.edu.cn (L.C.); jhyang1@jlnu.edu.cn (J.Y.); 2 Key Laboratory of Functional Materials Physics and Chemistry of the Ministry of Education, Jilin Normal University, Changchun 130103, China; 3 School of Materials Science and Engineering, Changchun University of Science and Technology, Changchun 130022, China; dlhan_1015@cust.edu.cn; 4 Technology Development Department, GLOBALFOUNDRIES (Singapore) Pte. Ltd., 60 Woodlands Industrial Park D, Street 2, Singapore 738406, Singapore; DANDAN.WANG@globalfoundries.com; 5 School of Environment and Safety Engineering, Jiangsu University, Zhenjiang 212013, China; lzy@mail.ujs.edu.cn; 6 United Microelect Corp. Ltd., 3 Pasir Ris Dr 12, Singapore 519528, Singapore

**Keywords:** Fe_3_O_4_ hollow microspheres, Fe_3_O_4_-Au magnetic nanocomposites, catalytic reduction, 4-nitrophenol

## Abstract

In this work, we report the enhanced catalytic reduction of 4-nitrophenol driven by Fe_3_O_4_-Au magnetic nanocomposite interface engineering. A facile solvothermal method is employed for Fe_3_O_4_ hollow microspheres and Fe_3_O_4_-Au magnetic nanocomposite synthesis via a seed deposition process. Complementary structural, chemical composition and valence state studies validate that the as-obtained samples are formed in a pure magnetite phase. A series of characterizations including conventional scanning/transmission electron microscopy (SEM/TEM), Mössbauer spectroscopy, magnetic testing and elemental mapping is conducted to unveil the structural and physical characteristics of the developed Fe_3_O_4_-Au magnetic nanocomposites. By adjusting the quantity of Au seeds coating on the polyethyleneimine-dithiocarbamates (PEI-DTC)-modified surfaces of Fe_3_O_4_ hollow microspheres, the correlation between the amount of Au seeds and the catalytic ability of Fe_3_O_4_-Au magnetic nanocomposites for 4-nitrophenol (4-NP) is investigated systematically. Importantly, bearing remarkable recyclable features, our developed Fe_3_O_4_-Au magnetic nanocomposites can be readily separated with a magnet. Such Fe_3_O_4_-Au magnetic nanocomposites shine the light on highly efficient catalysts for 4-NP reduction at the mass production level.

## 1. Introduction

Nowadays, numerous nitroaromatic compounds have been discharged into rivers with the overuse of dyes, explosives and pesticides in industry, causing serious water pollution [1]. Regrettably, 4-nitrophenol (4-NP) is the most typical toxic and refractory organic pollutant, attacking the eco-systems of both humans, as well as animals and consequently promoting various diseases [2]. Therefore, the degradation of 4-NP into non-toxic small molecules and in turn preventing harm has become a research hotspot in recent years [3,4].

Although it is difficult to degrade 4-NP due to the high stability and low solubility of 4-NP in water, many semiconductor nanomaterials (such as, ZnO [5], Cu_2_O [6] and TiO_2_ [7]) and various methods (e.g., typical photocatalysis and chemical catalysis [8]) have been developed to solve the pollution problems of 4-NP. Generally, the catalytic conversion of 4-NP to 4-aminophenol (4-AP) is an imperative process, using excess sodium borohydride as the reducing agent, not only because 4-AP is less toxic than 4-NP, but also because 4-AP is in demand on the market in many industrial fields [9]. Unfortunately, the reduction process of 4-NP to 4-AP is very slow by NaBH_4_ alone. Therefore, the use of catalysts is necessary to enable electron transfer from donor B^−^H_4_^−^ to acceptor 4-NP [10].

Most recently, noble metal nanocrystals have received both fundamental and practical attention owing to their potential applications in many fields such as ultrasensitive biosensing [11], imaging agents [12], photothermal therapy [13], catalysts [14], etc. Especially, Au nanoparticles (NPs) have been intensely explored because of their unique and tunable optical properties and high catalytic activities [15,16,17,18,19]. Compared to the semiconductor photocatalysts, noble metal catalysts possess higher catalytic activity with respect to organic pollutants [20,21,22,23,24]. Typically, Au NPs have been intensely employed for the catalytic reduction of a variety of organic pollutants [25]. However, Au NPs tend to agglomerate to form clusters due to their high surface area, reducing their intrinsic high catalytic activities [26,27]. To restrain the aggregation of Au NPs, considerable research efforts have been devoted to immobilization of Au NPs onto various supporting materials [28]. Several excellent reviews discussed that the stability of Au NPs could be significantly improved through solid supporting materials, such as carbon, silica, magnetic materials, and so on [29,30,31]. Among the various above-mentioned supports, iron oxides have received much more attention because they can be readily separated with a magnet and thereby possess the advantage of being magnetically recoverable and recyclable [32,33,34,35,36].

Theoretically, the shape and the size of the Fe_3_O_4_ play a deterministic role in defining the chemical and physical properties of Fe_3_O_4_ owing to the shape anisotropy [37]. For instance, the nanomaterials with a hollow structure have potential application prospects as catalysis and in biotechnology due to their higher load efficiency compared to other solid structures [38]. Therefore, Fe_3_O_4_ hollow microspheres have become the focus of study owing to their large specific surface area, excellent magnetic properties and hollow structural characteristics, allowing multiple different molecules to be loaded [39]. Notably, it is hard to directly attach Au NPs to the surfaces of the Fe_3_O_4_ hollow microspheres owing to the dissimilar nature of the two species’ surfaces. The use of the mediating “glue” layer of polymers supplying a certain kind of functional group is necessary, which not only can combine Au NPs with Fe_3_O_4_ hollow microspheres, but also enhance the stability, water solubility and the biocompatibility of nanomaterials [40]. For example, Wang et al. [41] reported that polyethyleneimine (PEI) could self-assemble on the surfaces of Fe_3_O_4_ to form a polymer shell, thus adhering to Au NPs via the abundant amine groups. However, the weak electrostatic interactions among positively-charged amino groups and negatively-charged Au NPs are unreliable, resulting in the partial separation of Au NPs from the polymers. Yan et al. [42] prepared bifunctional Fe_3_O_4_/Au nanocomposites by the direct reduction of HAuCl_4_ in the presence of carboxylate-functionalized Fe_3_O_4_ particles. Liu and co-workers [43] recently discovered that the bidentate ligands with two chelating sulfur groups such as the dithiocarbamates (DTCs) featuring carbodithioate (CS_2_) groups were more stable than other common ligands such as thiol and amino groups in adsorbing onto the surfaces of Au NPs, indicating that the PEI-DTC polymers may boost the Au NPs’ adhesive force onto Fe_3_O_4_ hollow microspheres’ surfaces.

**Figure 1:**
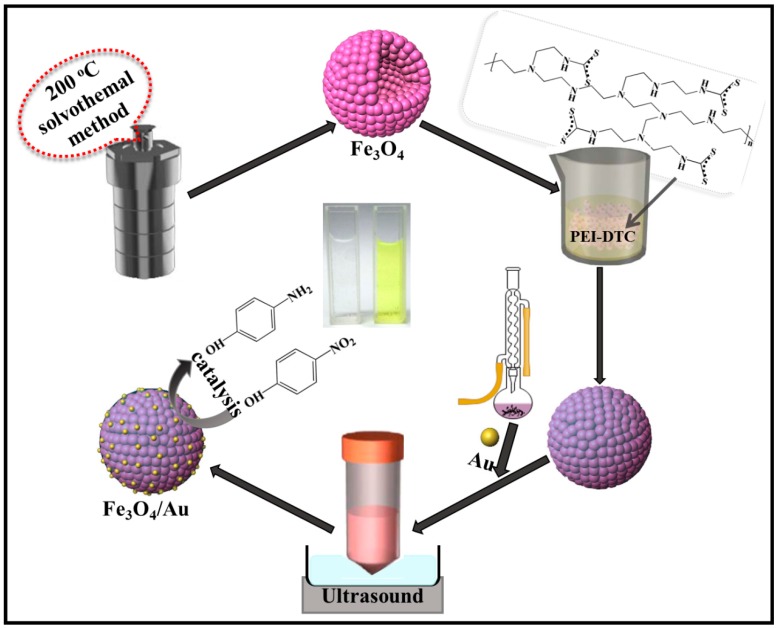
Schematic illustration of the fabrication process and catalytic application to 4-nitrophenol (4-NP) of Fe_3_O_4_-Au magnetic nanocomposites. PEI-DTC, polyethyleneimine-dithiocarbamate.

In this work, by adjusting the addition rounds of Au seeds, the amount of Au seeds coating the modified surfaces of Fe_3_O_4_ hollow microspheres can be well controlled, and the corresponding optical, magnetic and catalytic performances are investigated. Fe_3_O_4_-Au magnetic nanocomposites consisting of Fe_3_O_4_ hollow microspheres, PEI-DTC polymers and Au NPs are successfully utilized as effective nanocatalysts for the reduction of 4-NP to 4-AP by NaBH_4_. The aim of this study is to provide a new generation of magnetic nanocomposites with high catalytic efficiency and recyclable application to organic pollutants. The preparation and catalytic process to 4-NP of the Fe_3_O_4_-Au magnetic nanocomposites are shown in Figure 1.

## 2. Experimental Section

### 2.1. Materials’ Development

Chemicals, including methanol, polyethyleneimine (PEI, branched, MW ≈ 25,000 g/mol) and carbon disulfide (CS_2_), were purchased from Aladdin industrial Co., Ltd. (Shanghai, China). Iron chloride hydrate (FeCl_3_·6H_2_O) was purchased from Shanghai Macklin Biochemical Co., Ltd. (Shanghai, China). Ethylene glycol (EG), sodium dodecyl sulfate (SDS), sodium acetate (NaAc·3H_2_O), potassium hydroxide (KOH), sodium citrate dihydrate (Na_3_C_6_H_5_O_7_·2H_2_O), 4-nitrophenol (4-NP) and gold (III) chloride hydrate (HAuCl_4_·3H_2_O), were purchased from Sinopharm Chemical Reagent Co., Ltd. (Shanghai, China). The aforementioned reagents were analytical grade and were utilized without further purification.

### 2.2. Synthesis of Fe_3_O_4_ Hollow Microspheres

In a typical synthesis process for the Fe_3_O_4_ hollow microspheres, 1.62 g FeCl_3_·6H_2_O were dispersed in 60 mL EG into a beaker with mechanical stirring at room temperature. After 30 min, 2.70 g NaAc·3H_2_O and 0.1839 g SDS were added to the mixture. The mixture solution was vigorously stirred for 1 h until it became homogeneous. Then, the mixture was transferred to the Teflon-lined stainless steel autoclave, and it was kept at 200 °C for 12 h. After the samples were cooled to room temperature naturally, the products were obtained by centrifuging, sequentially rinsed with ethanol and deionized water for five times and subsequently dried under vacuum at 60 °C over night to obtain the Fe_3_O_4_ hollow microspheres.

### 2.3. Gold Seeds Synthesis

Approximately 80 mg Na_3_C_6_H_5_O_7_·2H_2_O were dissolved in 8 mL deionized water. Two milliliters of 58 mM HAuCl_4_·3H_2_O were diluted into 198 mL of deionized water with vigorous stirring. The mixture was subsequently heated under reflux at 105 °C. The well-prepared sodium citrates were steadily added dropwise. The reaction mixtures were sustained for 15 min under stirring and reflux, leading to a buff to burgundy color change. The mixture was eventually cooled down to room temperature naturally.

### 2.4. PEI-DTC Synthesis

Five hundred milligrams of PEI (0.02 mmol) and 650 mg KOH (11.6 mmol) were dissolved in 50 mL methanol under magnetic stirring with completely dissolved KOH. The solution was then purged with N_2_ for 15 s in order to exhaust the oxygen thoroughly, and 695 μL of CS_2_ (11.6 mmol) was added by dripping slowly into the mixed solution. PEI-DTC was obtained with stirring for 10 min with a solution color change to light yellow.

### 2.5. Fe_3_O_4_@PEI-DTC-Au Seeds’ Synthesis

In this step, the surfaces of Fe_3_O_4_ hollow microspheres were functionalized with PEI-DTC. Ten milligrams of Fe_3_O_4_ hollow microspheres were washed with methanol three times. PEI-DTC solution droplets were injected with a pipette under vortexing. The developed mixture was then kept for 1 h. The precipitate was gathered with a dedicated external magnet and rinsed with deionized water three times to remove the unnecessary PEI-DTC. Finally, Fe_3_O_4_@PEI-DTC NPs were re-dispersed in deionized water.

In comparison with counterpart bulk composites, equipped with a large surface area, the nanostructured materials demonstrate superior optical, magnetic and electrical characteristics [44,45,46,47,48]. The preparation of Fe_3_O_4_@PEI-DTC-Au seed nanocomposites is as follows. The as-obtained 10 mL of gold seed colloids were added dropwise into the 4 mL of Fe_3_O_4_@PEI-DTC NPs. Then, Fe_3_O_4_@PEI-DTC-Au nanocomposites were rinsed with deionized water and the same process repeated, and the sample was named as Fe_3_O_4_-Au 20 mL. Additional samples were prepared with the same conditions as Fe_3_O_4_-Au 20 mL, while the amount of gold seed colloids was changed to 40 and 60 mL, named as Fe_3_O_4_-Au 40 mL and Fe_3_O_4_-Au 60 mL, respectively.

### 2.6. Application of Fe_3_O_4_-Au Magnetic Nanocomposites for Catalytic Reduction of 4-NP

The catalytic ability of Fe_3_O_4_-Au magnetic nanocomposites was performed in a cuvette with 1 mg of Fe_3_O_4_-Au for the reduction of 4-NP (0.005 mol/L, 1 mL) in the presence of NaBH_4_ (0.2 mol/L, 1 mL). The UV-Vis spectrophotometer was used to record the catalytic reduction rate at different time intervals in the scanning range of 200–500 nm.

In order to study the recyclability of the prepared Fe_3_O_4_-Au magnetic nanocomposites, the samples were collected with a magnet from the reaction solution when the reduction process was completed. The obtained magnetic nanocomposites were rinsed with deionized water and ethanol three times and then repeated in the next reaction. The same catalytic reduction process to 4-NP was repeated six times.

### 2.7. Characterizations

The structure and morphology of the samples were characterized by X-ray diffractometer (XRD) (Rigaku D/Max-2500, Rigaku Corporation, Tokyo, Japan), Mössbauer spectrum (FAST Comtec Mössbauer system, FAST Comtec, Oberhaching, Germany), X-ray photoelectron spectroscopy (XPS) (Thermo Scientific ESCALAB 250Xi, Thermo Fisher Scientific, Waltham, MA, USA), field-emission scanning electron microscopy (FESEM) (JEOL JSM-7800F, JEOL Ltd., Tokyo, Japan) and transmission electron microscope (TEM) (JEOL 2100, JEOL Ltd., Tokyo, Japan). Ultraviolet-visible spectroscopy (UV-Vis) spectra and magnetic properties were measured with a Shimadzu UV 3600 spectrophotometer (Shimadzu Corporation, Tokyo, Japan) and a quantum design MPMS3 superconducting quantum interference device (SQUID) magnetometer (Quantum Design, Inc., San Diego, CA, USA), respectively.

## 3. Results and Discussion

### 3.1. X-ray Diffraction of the Fe_3_O_4_-Au Magnetic Nanocomposites

**Figure 2:**
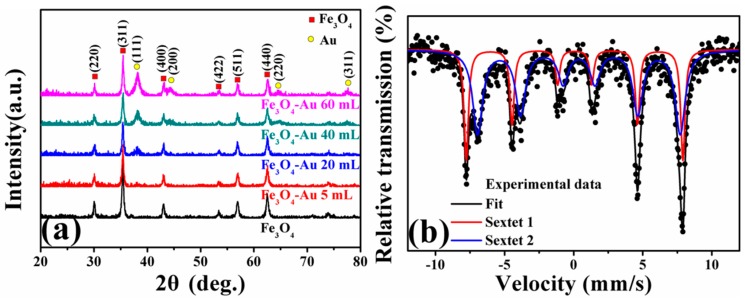
XRD patterns of the as-prepared pure Fe_3_O_4_ hollow microspheres and Fe_3_O_4_-Au magnetic nanocomposites with the different addition quantities of the gold seed colloids (Fe_3_O_4_-Au 5 mL, Fe_3_O_4_-Au 20 mL, Fe_3_O_4_-Au 40 mL and Fe_3_O_4_-Au 60 mL) (**a**); Mössbauer spectra of pure Fe_3_O_4_ hollow microsphere (**b**).

Figure 2a shows XRD patterns of the as-obtained pure Fe_3_O_4_ hollow microspheres and Fe_3_O_4_-Au magnetic nanocomposites with the various amounts of gold seed colloids added (Fe_3_O_4_-Au 5 mL, Fe_3_O_4_-Au 20 mL, Fe_3_O_4_-Au 40 mL and Fe_3_O_4_-Au 60 mL). As for the pure Fe_3_O_4_ hollow microspheres, the diffraction peaks located at 30.4°, 35.5°, 43.4°, 53.4°, 57.3° and 62.8° were indexed to (220), (311), (400), (422), (511) and (440) of Fe_3_O_4_ (Joint Committee on Powder Diffraction Standards, JCPDS 19-0629), respectively [21,49]. The average crystallite size of the pure Fe_3_O_4_ hollow microspheres was about 576.5 nm calculated by the Debye–Scherrer equation [50]. Four additional peaks of Fe_3_O_4_-Au magnetic nanocomposites at about 38.4°, 44.5°, 64.7° and 77.5° could be assigned into the (111), (200), (220) and (311) of Au (JCPDS 04-0784), respectively, suggesting the coexistence of Fe_3_O_4_ and Au [51,52]. Figure S1 presents the Pawley refinement of the XRD pattern of the pure Fe_3_O_4_ hollow microspheres. The residual weighted profile R-factor (Rwp) of the sample is 15.74%, which indicates that the sample is in agreement with the standard magnetite Fe_3_O_4_. In addition, it is well established that the area of the diffraction peak of XRD patterns is proportional to the contents of that phase in the mixture. Increasing addition times of gold seed colloids enhanced the diffraction peaks of Au, implying that the number of the gold seeds coating the surfaces of Fe_3_O_4_ hollow microspheres increases.

Because magnetite Fe_3_O_4_ and maghemite γ-Fe_2_O_3_ have nearly the same crystal structure of an inverse spinel type, it is difficult to differentiate them only based on the XRD results. The Mössbauer spectrum is an available characterization technique to distinguish magnetite from maghemite [53,54]. Mössbauer spectra of Fe_3_O_4_ hollow microspheres fitted with two sextets (Zeeman splitting patterns) are shown in Figure 2b. The acute and strong lines of the magnetic sextets show a characteristic double six-peak structure of magnetite Fe_3_O_4_ [55]. The hyperfine field is 48.5 and 45.5 Tesla, and the isomer shift is 0.603 and 0.287 mm/s, corresponding to Fe^2+^ and Fe^3+^ ions at octahedral interstitial sites and Fe^3+^ ions at tetrahedral interstitial sites, respectively. Table 1 lists the fitted Mössbauer parameters. All these results testify that the as-obtained sample is a pure magnetite Fe_3_O_4_ instead of maghemite γ-Fe_2_O_3_ and consistent with the results reported by Ghosh et al. [56]. In fact, the oxidation product of the magnetite Fe_3_O_4_ is either maghemite γ-Fe_2_O_3_ or hematite α-Fe_2_O_3_, which strongly depends on the oxidation temperature. Generally, a high temperature heat treatment is necessary to realize the phase transition of magnetite Fe_3_O_4_, which indirectly suggests that the structure of magnetite Fe_3_O_4_ is stable at room temperature [57,58].

### 3.2. Morphology of the Fe_3_O_4_-Au Magnetic Nanocomposites

**Figure 3:**
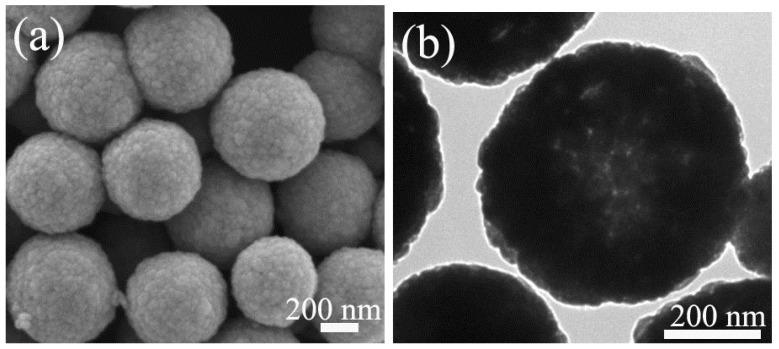
SEM images (**a**) and TEM images (**b**) of pure Fe_3_O_4_ hollow microspheres.

**Figure 4:**
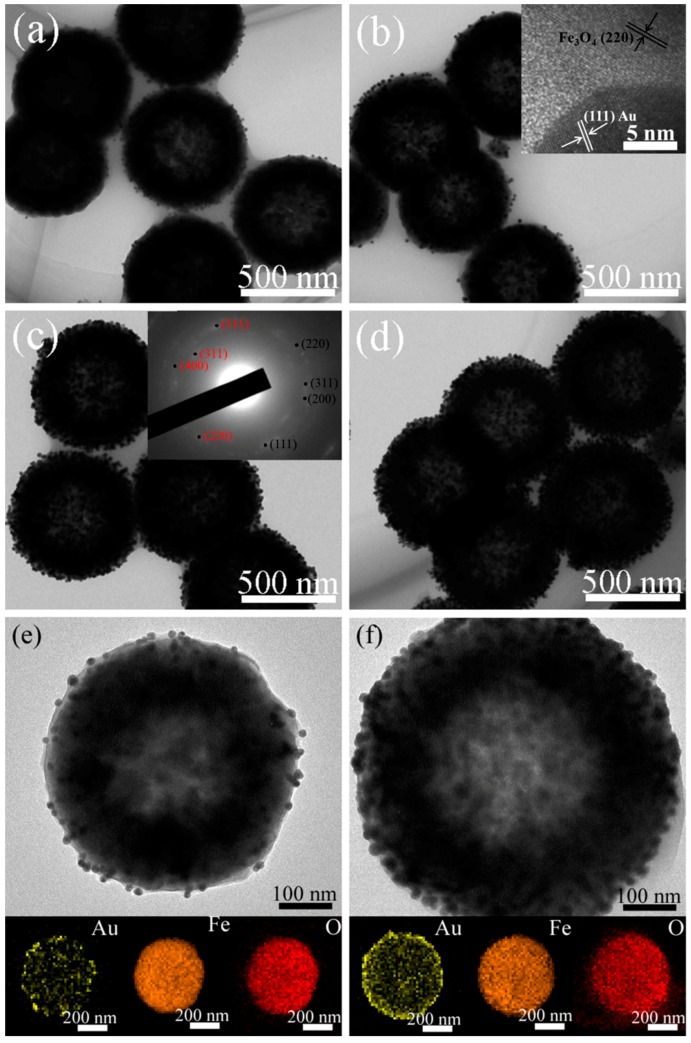
TEM images of Fe_3_O_4_-Au 5 mL (**a**) and Fe_3_O_4_-Au 20 mL with the HRTEM image (inset) (**b**); Fe_3_O_4_-Au 40 mL with the SAED pattern (inset) (**c**) and Fe_3_O_4_-Au 60 mL (**d**); TEM images of single Fe_3_O_4_-Au 5 mL (**e**) and Fe_3_O_4_-Au 60 mL (**f**) microspheres and corresponding EDS elemental mapping images (Au, Fe and O).

The specific structure and morphology of the developed Fe_3_O_4_-Au magnetic nanocomposites were studied by TEM and SEM. In particular, TEM measurement is an effective methodology for structure research in materials science and materials engineering [59,60,61,62,63,64,65,66,67,68,69,70,71]. Figure 3 displays SEM and TEM images of pure Fe_3_O_4_ hollow microspheres. It can be observed that the pure Fe_3_O_4_ hollow microspheres are spherical shape. The average crystallite size is about 576 nm, and the samples show the narrow size distribution, as shown in Figure S2. TEM and SEM images of Fe_3_O_4_-Au 5 mL, Fe_3_O_4_-Au 20 mL, Fe_3_O_4_-Au 40 mL and Fe_3_O_4_-Au 60 mL are presented in Figure 4 and Figure S3. The as-prepared Fe_3_O_4_-Au magnetic nanocomposites consist of the Fe_3_O_4_ hollow microspheres and the gold seeds of about 16 nm in diameter. Clearly, the gold seeds are randomly and homogeneously attached to the surfaces of the Fe_3_O_4_ hollow microspheres. Gold seeds are seen black, and Fe_3_O_4_ hollow microspheres present a light color in the TEM images, attributed to the higher electron density of Au compared to that of Fe_3_O_4_ [72]. It can be easily observed from the high-resolution TEM (HRTEM) image in the inset of Figure 4b that the interplanar distance values are 0.204 and 0.217 nm, matching the (220) lattice plane of Fe_3_O_4_ and the (111) lattice plane of Au, respectively [73]. The selected area electron diffraction (SAED) pattern of Fe_3_O_4_-Au 40 mL is presented in the inset of Figure 4c, consisting of both the (220), (311), (400) and (511) diffraction rings of Fe_3_O_4_ and the (111), (200), (220) and (311) diffraction rings of Au [43]. The HRTEM and SAED results further verify the coexistence of Fe_3_O_4_ and Au. An interesting finding is that when increasing Au seed colloids’ addition times, the gold seed contents on Fe_3_O_4_ surfaces are further boosted, which is consistent with the aforementioned XRD data. This is supported by the following elemental mapping images. 

Figure 4e,f shows the TEM images of single Fe_3_O_4_-Au 5 mL and Fe_3_O_4_-Au 60 mL microspheres and corresponding energy-dispersive X-ray (EDS) mapping images of Au, Fe and O elements [74,75]. It could be clearly found that the Fe_3_O_4_ hollow microspheres have hollow internal structures, and Au seeds are homogeneously attached to the surfaces of the Fe_3_O_4_ hollow microspheres. In addition, compared with Fe_3_O_4_-Au 5 mL, the amount of gold seeds on the surfaces of Fe_3_O_4_ for Fe_3_O_4_-Au 60 mL increases significantly.

### 3.3. Optical Properties of the Fe_3_O_4_-Au Magnetic Nanocomposites

**Figure 5:**
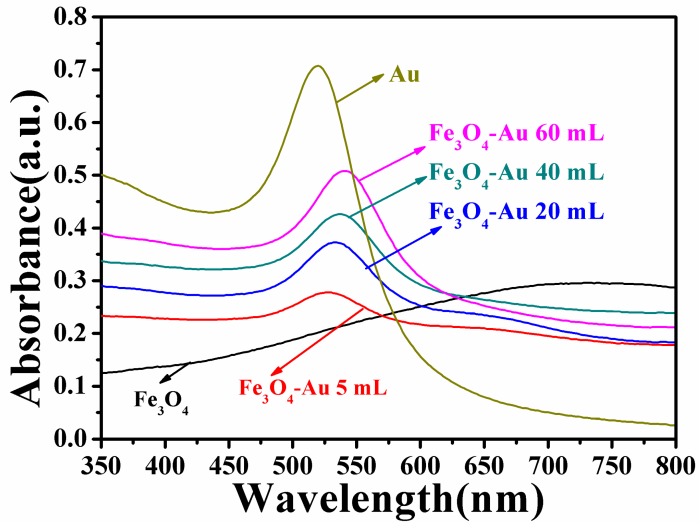
UV-Vis absorption spectra of pure Fe_3_O_4_ hollow microspheres, Au seed colloids, Fe_3_O_4_-Au 5 mL, Fe_3_O_4_-Au 20 mL, Fe_3_O_4_-Au 40 mL and Fe_3_O_4_-Au 60 mL magnetic nanocomposites.

UV-Vis spectroscopy studies are carried out to investigate the optical properties of pure Fe_3_O_4_ hollow microspheres and Fe_3_O_4_-Au magnetic nanocomposites. Figure 5 presents the UV-Vis spectra of pure Fe_3_O_4_ hollow microspheres, Au seed colloids, Fe_3_O_4_-Au 5 mL, Fe_3_O_4_-Au 20 mL, Fe_3_O_4_-Au 40 mL and Fe_3_O_4_-Au 60 mL magnetic nanocomposites. No absorption peak is observed for the pure Fe_3_O_4_ hollow microspheres in the visible wavelength span [34,76]. The Au gold seed colloids display a characteristic surface plasmon resonance (SPR) peak at ~520 nm on account of the coherent excitation of the free electrons within the conduction band [77]. In addition, all the Fe_3_O_4_-Au magnetic nanocomposites show a clear characteristic SPR band of gold nanostructures. Interestingly, the absorption peak of Fe_3_O_4_-Au magnetic nanocomposites is broadened and shows a slight red shift compared with that of pure Au seed colloids. The more Au seeds are deposited on Fe_3_O_4_ hollow microspheres’ surfaces, the more evident the red shift of the absorption peak is. The absorption band shows a red-shift from 526 nm for Fe_3_O_4_-Au 5 mL to 540 nm for Fe_3_O_4_-Au 60 mL. This red shift of the SPR absorption band can be ascribed to plasmon hybridization and the surface plasmon coupling of neighboring Au seeds deposited on Fe_3_O_4_ hollow microspheres [52,78].

### 3.4. XPS of the Fe_3_O_4_-Au Magnetic Nanocomposites

**Figure 6:**
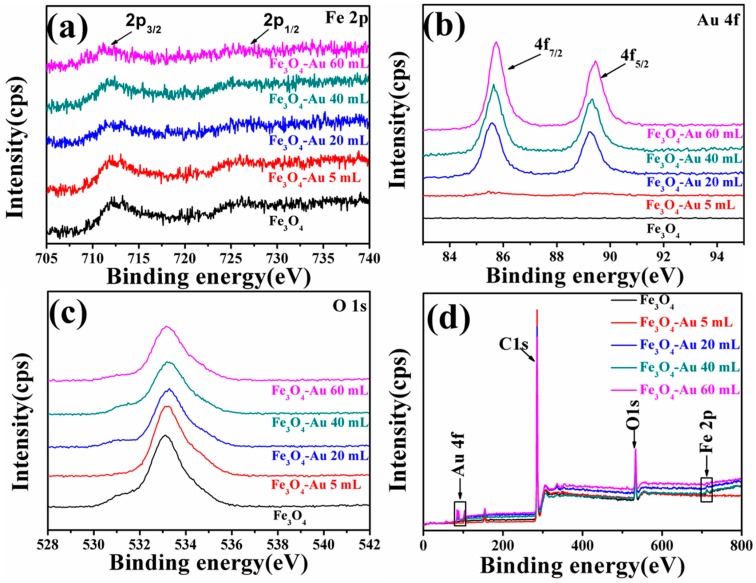
XPS spectra of the as-obtained Fe_3_O_4_, Fe_3_O_4_-Au 5 mL, Fe_3_O_4_-Au 20 mL, Fe_3_O_4_-Au 40 mL and Fe_3_O_4_-Au 60 mL: the Fe 2p binding energies (**a**); the Au 4f binding energies (**b**); the O1s binding energies (**c**) and XPS survey spectra (**d**).

XPS was applied to characterize the elemental chemical states and chemical compositions of the pure Fe_3_O_4_ hollow microspheres and Fe_3_O_4_-Au magnetic nanocomposites. The C 1s peak at 284.8 eV is applied as the reference for charge correction [79]. The XPS results of Fe_3_O_4_, Fe_3_O_4_-Au 5 mL, Fe_3_O_4_-Au 20 mL, Fe_3_O_4_-Au 40 mL and Fe_3_O_4_-Au 60 mL are shown in Figure 6. Figure 6a is the high-resolution scans of the XPS spectra for Fe 2p. The binding energy of pure Fe_3_O_4_ hollow microspheres is assessed at 711.2 and 724.5 eV by using Gaussian-Lorentzian fitting and assigned to the Fe 2p_3/2_ and Fe 2p_1/2_ of magnetite Fe_3_O_4_, respectively [80]. As for Fe_3_O_4_-Au 20 mL (Figure 6b), the peak doublet appearing at 83.9 and 87.9 eV with a spin orbit splitting of ~4 eV is assigned to Au 4f_7/2_ and Au 4f_5/2_, respectively [81]. It is important to highlight that with the increase of the quantity of the Au seeds on the Fe_3_O_4_ surfaces, the intensity of Fe 2p decreases and the intensity of Au 4f increases, because the intensity of XPS spectra is proportional to the atomic concentration and the atomic sensitivity factor [82]. Meanwhile, Fe 2p-associated peaks shift toward lower binding energy, while Au 4f peaks shift toward the opposite binding energy side. The shift in binding energy provides evidence of an interaction between Au and Fe_3_O_4_. The chemical shifts in the Fe 2p peaks and Au 4f peaks are probably caused by the transfer of charge carriers at the metal-oxide interfaces [83,84,85]. As shown in Figure 6c, the O 1s spectrum of pure Fe_3_O_4_ exhibits only a single peak at 532.8 eV, and no obvious shift of peak position is observed [86]. The survey scan results demonstrate that all the indexed peaks correspond to those of Fe, Au, O and C (Figure 6d).

### 3.5. Magnetic Properties of the Fe_3_O_4_-Au Magnetic Nanocomposites

**Figure 7:**
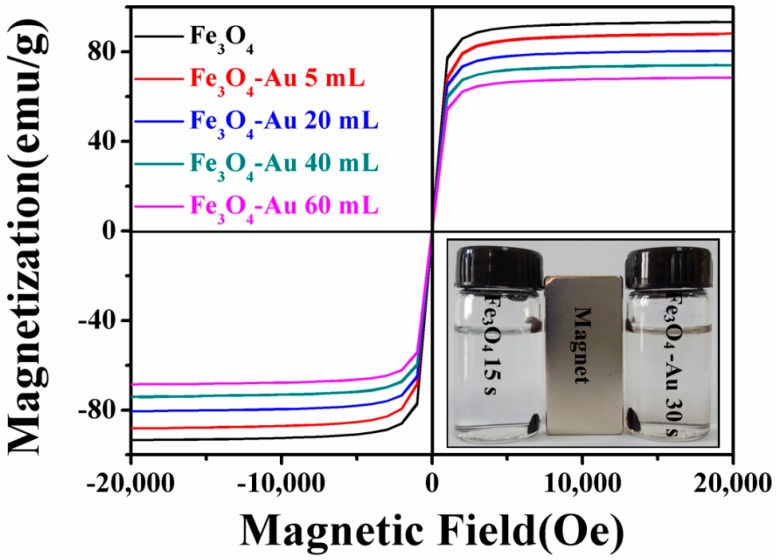
Magnetic hysteresis (M-H) loops of Fe_3_O_4_, Fe_3_O_4_-Au 5 mL, Fe_3_O_4_-Au 20 mL, Fe_3_O_4_-Au 40 mL and Fe_3_O_4_-Au 60 mL. The inset is the photograph of pure Fe_3_O_4_ hollow microspheres and Fe_3_O_4_-Au 60 mL magnetic nanocomposites in deionized water after using a magnet.

The magnetic properties of the obtained samples were measured using SQUID. The magnetization versus magnetic field (M-H) loops of pure Fe_3_O_4_ hollow microspheres and Fe_3_O_4_-Au magnetic nanocomposites are presented in Figure 7. The M-H loops of all the samples are S-shaped curves and exhibit negligible coercivity and remanence, which is the typical characteristic of superparamagnetic nanomaterials [87]. Moreover, in order to confirm the superparamagnetism of the as-obtained sample, the temperature-dependent magnetization (M-T) on pure Fe_3_O_4_ hollow microspheres was measured under zero-field-cooled (ZFC) and 1000 Oe field-cooled (FC) conditions (Figure S4). With the increase of temperature, the magnetization increases to a maximum at the blocking temperature and then decreases, consistent with the superparamagnetic behavior [88]. The saturation magnetization (Ms) of pure Fe_3_O_4_ hollow microspheres reaches up to 93.2 emu/g, but the Ms value of Fe_3_O_4_-Au 5 mL reduces to 88.1 emu/g. Further coating with the gold seeds results in a continuous decrease of the Ms value. As for Fe_3_O_4_-Au 60 mL, the Ms value is only 68.6 emu/g. The gradual decrease in the Ms value can be ascribed to the increase in the weight ratio of Au seeds to Fe_3_O_4_ hollow microspheres or the diamagnetic contribution of the Au seeds deposited onto the surfaces of the Fe_3_O_4_ hollow microspheres [89,90,91]. It must also be mentioned that although Ms values decrease owing to the introduction of Au seeds, the Fe_3_O_4_-Au magnetic nanocomposites retain the strong magnetic responsivity and can be easily magnetically collected from aqueous solution by the magnet (as shown in the inset of Figure 7), which is beneficial to their economic and reusable applications.

### 3.6. Catalytic Activity of Fe_3_O_4_-Au Magnetic Nanocomposites to 4-NP

**Figure 8:**
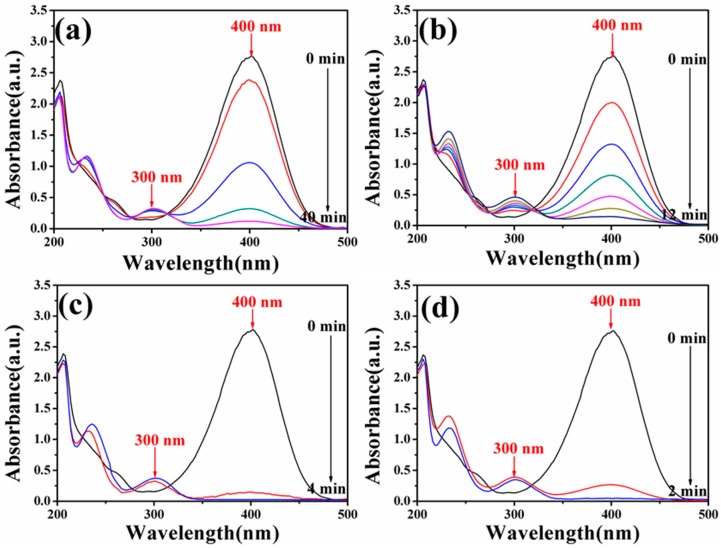
UV-Vis absorption spectra of 4-NP after reduction catalyzed by Fe_3_O_4_-Au 5 mL (**a**); Fe_3_O_4_-Au 20 mL (**b**); Fe_3_O_4_-Au 40 mL (**c**) and Fe_3_O_4_-Au 60 mL (**d**).

**Figure 9:**
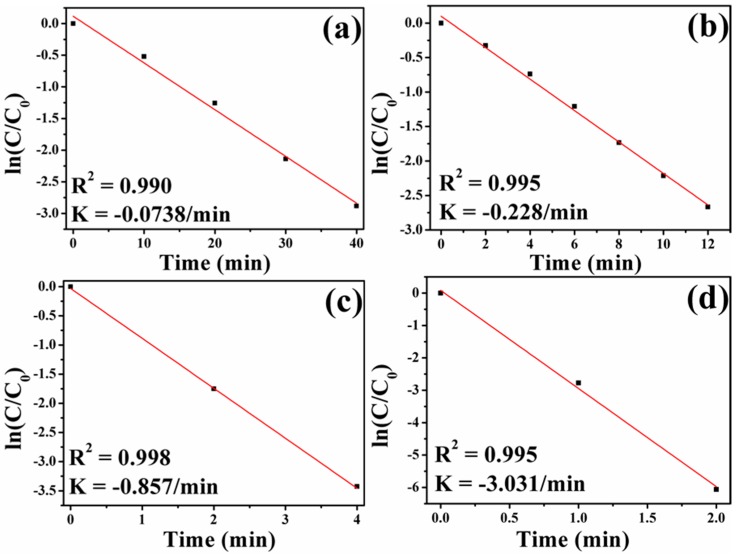
Plots of ln(C/C_0_) against reaction time: Fe_3_O_4_-Au 5 mL (**a**); Fe_3_O_4_-Au 20 mL (**b**); Fe_3_O_4_-Au 40 mL (**c**) and Fe_3_O_4_-Au 60 mL (**d**).

To study the catalytic performance of Fe_3_O_4_-Au magnetic nanocomposites to organic pollutants, the catalytic reduction of 4-NP in the presence of excess NaBH_4_ was selected as a model reaction. As shown in Figure S5, the original aqueous 4-NP solution has a maximum absorption peak at 317 nm. If the catalysts are absent, the absorption peak at 317 nm is unchanged even after a couple of days [20]. Once freshly obtained NaBH_4_ solution is added, the absorption peak at 317 nm shifts to 400 nm due to the formation of 4-nitrophenolate ions, and meanwhile, the color of the mixture changes from light yellow to dark yellow [92]. However, the reduction reaction cannot be produced without gold seeds even in the presence of excess NaBH_4_, as can be testified in Figure S6. There is almost no change in the intensity at 400 nm after 480 min, indicating that the addition of pure Fe_3_O_4_ hollow microspheres has little influence on the reduction of 4-NP. Figure 8 exhibits UV-Vis absorption spectra of 4-NP catalyzed by the different Fe_3_O_4_-Au magnetic nanocomposites (Fe_3_O_4_-Au 5 mL, Fe_3_O_4_-Au 20 mL, Fe_3_O_4_-Au 40 mL and Fe_3_O_4_-Au 60 mL) in the presence of NaBH_4_ at a certain time interval. When Fe_3_O_4_-Au magnetic nanocomposites are introduced into the reaction system, the absorption peak of all the samples at 400 nm decreases in intensity. At the same time, a new peak located at 300 nm appears and increases concomitantly in intensity with the reaction time, which is attributed to the formation of 4-AP, the corresponding product in the reduction of 4-NP. The whole peak at 400 nm disappears after 40, 12, 4 and 2 min catalyzed by Fe_3_O_4_-Au 5 mL, Fe_3_O_4_-Au 20 mL, Fe_3_O_4_-Au 40 mL and Fe_3_O_4_-Au 60 mL, respectively. The color of the solution transforms from yellow to colorless, demonstrating that the 4-NP is completely changed into 4-AP and no other byproducts are generated. The corresponding pseudo-first-order plots of ln(C/C_0_) versus reaction time over different Fe_3_O_4_-Au magnetic nanocomposites are portrayed in Figure 9. The concentration of NaBH_4_ is identified as a constant because it is much more excessive compared to that of 4-NP. The ratio of C (the concentration of 4-NP at reaction time t) to C_0_ (the initial concentration of 4-NP) is obtained from the relative intensity of respective absorbance (A_t_/A_0_) at 400 nm. The linear relationship of ln(C/C_0_) against reaction time represents that the reduction of 4-NP by the Fe_3_O_4_-Au magnetic nanocomposites follows the pseudo first order kinetics. The rate constant (k) can be calculated by a liner plot of ln(C/C_0_) vs. reduction time. The rate constants of 4-NP are 0.0738, 0.228, 0.857 and 3.031/min using Fe_3_O_4_-Au 5 mL, Fe_3_O_4_-Au 20 mL, Fe_3_O_4_-Au 40 mL and Fe_3_O_4_-Au 60 mL as nanocatalysts, respectively. Obviously, the rate constants for 4-NP reduction increase when increasing Au seed addition rounds. It is reasonable that if the noble metal nanomaterials’ loading is increased, the mass fraction of the noble metal nanomaterials is higher in the final samples, and thus, a better catalytic performance should be accomplished [93]. We thus propose that the catalytic capability of Fe_3_O_4_-Au magnetic nanocomposites could be greatly improved when more gold seeds cover the surfaces of the Fe_3_O_4_ hollow microspheres.

**Figure 10:**
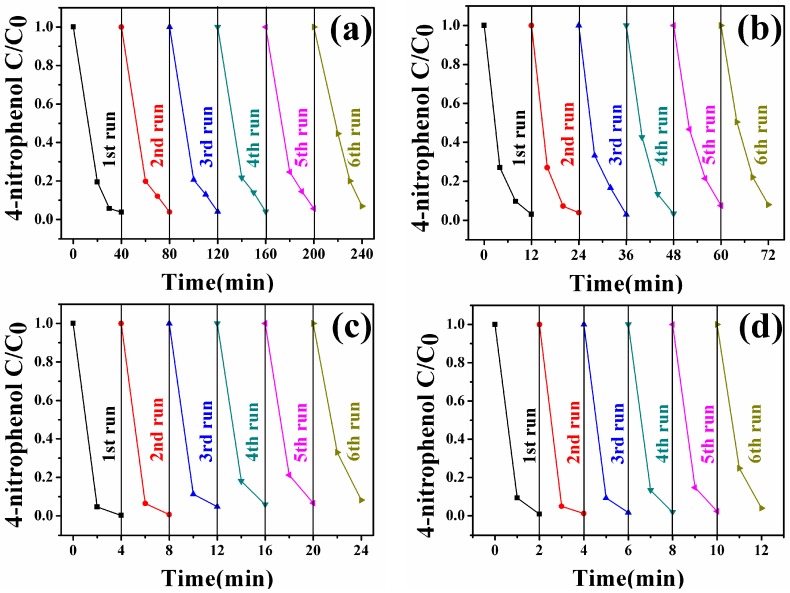
Reusability of Fe_3_O_4_-Au 5 mL (**a**); Fe_3_O_4_-Au 20 mL (**b**); Fe_3_O_4_-Au 40 mL (**c**) and Fe_3_O_4_-Au 60 mL (**d**) for catalytic reduction of 4-NP.

To implement the practical application of Fe_3_O_4_-Au magnetic nanocomposites, their stability and the recyclability are essential. All the Fe_3_O_4_-Au magnetic nanocomposites were separated magnetically and reused after catalytic reduction of 4-NP. As shown in Figure 10, all the samples can be successfully reused for at least six reaction runs for the catalytic reduction of 4-NP, elaborating that the Fe_3_O_4_-Au magnetic nanocomposites possess the excellent stability and can serve as recoverable and efficient nanocatalysts of the organic pollutants.

## 4. Conclusions

In summary, Fe_3_O_4_-Au magnetic nanocomposites were prepared by a well-developed seed deposition method. Structural analyses confirmed that the as-prepared Fe_3_O_4_-Au magnetic nanocomposites possessed a high surface area and that the gold seeds were homogeneously deposited onto the Fe_3_O_4_ hollow microspheres’ surfaces. The increase of the quantity of the gold seeds attached to the surfaces of the Fe_3_O_4_ hollow microspheres results in Fe 2p peaks shifting toward a lower binding energy and Au 4f peaks shifting towards a higher binding energy. Although the Ms value decreases with the increase of the gold seed amount deposited on the Fe_3_O_4_ surfaces, the samples retain the strong magnetic responsivity and can be easily collected by a magnet. The pseudo-first-order kinetics are used to calculate the rate constant of 4-NP, and the rate constants for the 4-NP reduction increase when increasing the added amount of gold seeds. Furthermore, Fe_3_O_4_-Au magnetic nanocomposites can serve as recyclable nanocatalysts for 4-NP. Therefore, Fe_3_O_4_-Au magnetic nanocomposites can be excellent nanocatalysts for the catalytic reduction of organic pollutants in the treatment of waste water.

## Figures and Tables

**Table 1:** Mössbauer spectrum parameters of pure Fe_3_O_4_ hollow microspheres: IS is the isomer shift, QS the quadrupole splitting, HIN the hyperfine field, HWHM the half width at half maximum and AREA the relative absorption area.

Composition	IS (mm/s)	QS (mm/s)	HIN (T)	HWHM (mm/s)	AREA (%)
A	0.287	0.015	48.5	0.186	34.3
B	0.603	0.012	45.5	0.433	65.7

## Supplementary Materials

The following are available online at http://www.mdpi.com/2079-4991/8/5/353/s1. Figure S1: Pawley refinement of the XRD pattern of the pure Fe_3_O_4_ hollow microspheres. Red dots, blue lines and olive bars represent the experimental data, the calculated date and the peak position of the sample, respectively. The bottom line in black shows the different experimental-calculated data, Figure S2: Particle size distribution obtained from the analysis of the TEM images for pure Fe_3_O_4_ hollow microspheres, Figure S3: SEM and corresponding EDS images of the as-prepared Fe_3_O_4_-Au nanocomposites with the different addition quantities of the gold seed colloids (Fe_3_O_4_ (a, b and c), Fe_3_O_4_-Au 5 mL (d, e and f), Fe_3_O_4_-Au 20 mL (g, h and i), Fe_3_O_4_-Au 40 mL (j, k and l) and Fe_3_O_4_-Au 60 mL (m, n and o), Figure S4: ZFC and FC curves of pure Fe_3_O_4_ hollow microspheres under an applied field of 1000 Oe, Figure S5: UV-Vis absorption spectra of 4-NP before (red line) and after adding NaBH_4_ solution (black line), Figure S6: UV-Vis absorption spectra of 4-NP catalyzed by pure Fe_3_O_4_ hollow microspheres in presence of NaBH_4_ before and after 480 min.

## References

[1] Zhang X.F., Zhu X.Y., Feng J.J., Wang A.J. (2018). Solvothermal synthesis of N-doped graphene supported PtCo nanodendrites with highly catalytic activity for 4-nitrophenol reduction. Appl. Surf. Sci..

[2] Bordbar M., Negahdar N., Nasrollahzadeh M. (2018). Melissa Officinalis L. leaf extract assisted green synthesis of CuO/ZnO nanocomposite for the reduction of 4-nitrophenol and Rhodamine B. Sep. Purif. Technol..

[3] Nag S., Pramanik A., Chattopadhyay D., Bhattacharyya M. (2017). Green-fabrication of gold nanomaterials using Staphylococcus warneri from Sundarbans estuary: An effective recyclable nanocatalyst for degrading nitro aromatic pollutants. Environ. Sci. Pollut. Res..

[4] Meziane D., Benadda-Kordjani A., Nezzal G., Benammar S., Djadoun A. (2017). Para-nitrophenol reduction on solvothermally prepared cobalt@silica core-shell catalysts. React. Kinet. Mech. Catal..

[5] He L.L., Tong Z.F., Wang Z.H., Chen M., Huang N., Zhang W. (2017). Effects of calcination temperature and heating rate on the photocatalytic properties of ZnO prepared by pyrolysis. J. Colloid Interface Sci..

[6] Liu L., Yang W., Sun W., Li Q., Shang J.K. (2015). Creation of Cu_2_O@TiO_2_ composite photocatalysts with p-n heterojunctions formed on exposed Cu_2_O facets, their energy band alignment study, and their enhanced photocatalytic activity under visible light illumination. ACS Appl. Mater. Interfaces.

[7] Andreou D., Iordanidou D., Tamiolakis I., Armatas G.S., Lykakis I.N. (2016). Reduction of nitroarenes into aryl amines and *n*-aryl hydroxylamines via activation of NaBH_4_ and ammonia-borane complexes by Ag/TiO_2_ catalyst. Nanomaterials.

[8] Zhao X., Wang Y., Feng W.H., Lei H.T., Li J. (2017). Preparation of Cu(II) porphyrin-TiO_2_ composite in one-pot method and research on photocatalytic property. RSC Adv..

[9] Liu Y., Zhang Y.Y., Kou Q.W., Chen Y., Han D.L., Wang D.D., Lu Z.Y., Chen L., Yang J.H., Xing S. (2018). Eco-friendly seeded Fe_3_O_4_-Ag nanocrystals: A new type of highly efficient and low cost catalyst for methylene blue reduction. RSC Adv..

[10] Shah M.T., Balouch A., Sirajuddin, Pathan A.A., Abdullah, Mahar A.M., Sabir S., Khattak R., Umar A.A. (2017). SiO_2_ caped Fe_3_O_4_ nanostructures as an active heterogeneous catalyst for 4-nitrophenol reduction. Microsyst. Technol..

[11] Svedendahl M., Verre R., Kall M. (2014). Refractometric Biosensing Based on Optical Phase Flips in Sparse and Short-Range-Ordered Nanoplasmonic Layers. Light Sci. Appl..

[12] Zhu Z., Bai B., You O., Li Q., Fan S. (2015). Fano resonance boosted cascaded optical field enhancement in a plasmonic nanoparticle-in-cavity nanoantenna array and its SERS application. Light Sci. Appl..

[13] Wang P., Wang Y., Tong L. (2013). Graphene-doped polymer nanofibers for low-threshold nonlinear optical waveguiding. Light Sci. Appl..

[14] Li X., Xing L., Zheng K., Wei P., Du L., Shen M., Shi X. (2017). Formation of gold nanostar-coated hollow mesoporous silica for tumor multimodality imaging and photothermal therapy. ACS Appl. Mater. Interfaces.

[15] Aslam U., Chavez S., Linic S. (2017). Controlling energy flow in multimetallic nanostructures for plasmonic catalysis. Nat Nano.

[16] Zhang Q., Wang H. (2014). Facet-Dependent Catalytic Activities of Au Nanoparticles Enclosed by High-Index Facets. ACS Catal..

[17] Karabchevsky A., Mosayyebi A., Kavokin A.V. (2016). Tuning the chemiluminescence of a luminol flow using plasmonic nanoparticles. Light Sci. Appl..

[18] Linnenbank H., Grynko Y., Forstner J., Linden S. (2016). Second harmonic generation spectroscopy on hybrid plasmonic/dielectric nanoantennas. Light Sci. Appl..

[19] Blum O., Shaked N.T. (2015). Prediction of photothermal phase signatures from arbitrary plasmonic nanoparticles and experimental verification. Light Sci. Appl..

[20] Gangarapu M., Sarangapany S., Veerabhali K.K., Devipriya S.P., Arava V.B.R. (2017). A high-performance catalytic and recyclability of phyto-synthesized silver nanoparticles embedded in natural polymer. J. Clust. Sci..

[21] Jin C.J., Qu Y., Wang M.G., Han J., Hu Y.M., Guo R. (2016). Aqueous solution-based Fe_3_O_4_ seed-mediated route to hydrophilic Fe_3_O_4_-Au janus nanoparticles. Langmuir.

[22] Navalón S., García H. (2016). Nanoparticles for Catalysis. Nanomaterials.

[23] Maham M., Nasrollahzadeh M., Sajadi S.M., Nekoei M. (2017). Biosynthesis of Ag/reduced graphene oxide/Fe_3_O_4_ using Lotus Garcinii leaf extract and its application as a recyclable nanocatalyst for the reduction of 4-nitrophenol and organic dyes. J. Colloid Interface Sci..

[24] Jia L., Zhou T., Xu J., Li F.H., Xu Z.Q., Zhang B.B., Guo S.L., Shen X.K., Zhang W.S. (2017). AuPd bimetallic nanocrystals embedded in magnetic halloysite nanotubes: Facile synthesis and catalytic reduction of nitroaromatic compounds. Nanomaterials.

[25] Xiao Z.Y., Zhai S.R., Ma X.P., Zhao Z.Y., Wang X., Bai H., An Q.D. (2017). Monolithic Cu/C hybrid beads with well developed porosity for reduction of 4-nitrophenol to 4-aminophenol. New J. Chem..

[26] Zhang K.H., Wang C.W., Rong Z., Xiao R., Zhou Z., Wang S.Q. (2017). Silver coated magnetic microflowers as an efficient and recyclable Catalyst for catalytic reduction. New J. Chem..

[27] Sun J., Chen L. (2017). Superparamagnetic POT/Fe_3_O_4_ nanoparticle composites with supported Au nanoparticles as recyclable high-performance nanocatalysts. Mater. Today Chem..

[28] Wang D.M., Duan H.C., Lü J.H., Lü C.L. (2017). Fabrication of thermo-responsive polymer functionalized reduced graphene oxide@Fe_3_O_4_@Au magnetic nanocomposites for enhanced catalytic applications. J. Mater. Chem. A.

[29] Rath P.C., Saikia D., Mishra M., Kao H.M. (2018). Exceptional catalytic performance of ultrafine Cu_2_O nanoparticles confined in cubic mesoporous carbon for 4-nitrophenol reduction. Appl. Surf. Sci..

[30] Meng G.H., Zhang X.Y., Liu C., Wu J.N., Guo X.H., Liu Z.Y. (2017). Ag quantum dot/montmorillonite compositeswith fluorescent properties: An efficient catalyst. Res. Chem. Intermed..

[31] Govan J., Gun’ko Y.K. (2014). Recent advances in the application of magnetic nanoparticles as a support for homogeneous catalysts. Nanomaterials.

[32] Liu Y., Zhang Y.Y., Kou Q.W., Chen Y., Sun Y.T., Han D.L., Wang D.D., Lu Z.Y., Chen L., Yang J.H., Xing S.G.Z. (2018). Highly Efficient, Low-Cost, and Magnetically Recoverable FePt–Ag Nanocatalysts Towards Green Reduction of Organic Dyes. Nanomaterials.

[33] Wysocka I., Kowalska E., Trzciński K., Łapiński M., Nowaczyk G., Zielińska-Jurek A. (2018). UV-Vis-Induced Degradation of Phenol over Magnetic Photocatalysts Modified with Pt, Pd, Cu and Au Nanoparticles. Nanomaterials.

[34] Pardo I.R., Pons M.R., Heredia A.A., Usagre J.V., Ribera A., Galian R.E., Prieto J.P. (2017). Fe_3_O_4_@Au@mSiO_2_ as enhancing nanoplatform for rose bengal photodynamic activity. Nanoscale.

[35] Wang Y.S., Wang Y., Xia H., Wang G., Zhang Z.Y., Han D.D., Lv C., Feng J., Sun H.B. (2016). Preparation of Fe_3_O_4_-Au-GO nanocomposite for simultaneous treatment of oil/water separation and dye decomposition. Nanoscale.

[36] Guo R., Jiao T.F., Xing R.R., Chen Y., Guo W.C., Zhou J.X., Zhang L.X., Peng Q.M. (2017). Hierarchical AuNPs-Loaded Fe_3_O_4_/Polymers Nanocomposites Constructed by Electrospinning with Enhanced and magnetically recyclable catalytic capacities. Nanomaterials.

[37] Tedsree K., Temnuch N., Sriplai N., Pinitsoontorn S. (2017). Ag modified Fe_3_O_4_@TiO_2_ magnetic core-shell nanocomposites for photocatalytic degradation of methylene blue. Mater. Today.

[38] Yang J.H., Kou Q.W., Liu Y., Wang D.D., Lu Z.Y., Chen L., Zhang Y.Y., Wang Y.X., Zhang Y.J., Han D.L. (2017). Effects of amount of benzyl ether and reaction time on the shape and magnetic properties of Fe_3_O_4_ nanocrystals. Powder Technol..

[39] Duan L.F., Jia S.S., Wang T.H., Xue B., Wang Y.Q., Zhao L.J. (2011). Synthesis and Characterization of hollow Fe_3_O_4_ submicrospheres by a Simple Solvothermal Synthesis. Met. Mater. Int..

[40] Hu Y., Yang J., Wei P., Li J.C., Ding L., Zhang G.X., Shi X.Y., Shen M.W. (2015). Facile synthesis of hyaluronic acid-modified Fe_3_O_4_/Au composite nanoparticles for targeted dual mode MR/CT imaging of tumors. J. Mater. Chem. B.

[41] Wang C.W., Li P., Wang J.F., Rong Z., Pang Y.F., Xu J.W., Dong P.T., Xiao R., Wang S.Q. (2015). Polyethylenimine-interlayered core-shell-satellite 3D magnetic microspheres as versatile SERS substrate. Nanoscale.

[42] Yan F., Sun R.Y. (2014). Facile Synthesis of Bifunctional Fe_3_O_4_/Au Nanocomposite and Their Application in Catalytic Reduction of 4-Nitrophenol. Mater. Res. Bull..

[43] Liu Y., Kou Q.W., Wang D.D., Chen L., Sun Y.T., Lu Z.Y., Zhang Y.Y., Wang Y.X., Yang J.H., Xing S. (2017). Rational synthesis and tailored optical and magnetic Characteristics of Fe_3_O_4_-Au composite nanoparticles. J. Mater. Sci..

[44] Xing G.Z., Wang Y., Wong J.I., Shi Y.M., Huang Z.X., Li S., Yang H.Y. (2014). Hybrid CuO/SnO_2_ nanocomposites: Towards cost-effective and high performance binder free lithium ion batteries anode materials. Appl. Phys. Lett..

[45] Xing G.Z., Wang D.D., Cheng C.J., He M., Li S., Wu T. (2013). Emergent ferromagnetism in ZnO/Al_2_O_3_ core-shell nanowires: Towards oxide spinterfaces. Appl. Phys. Lett..

[46] Sun Y., Li Q. (2016). Research of zinc oxide quantum dot light-emitting diodes based on preparation of chemical solutions. Chin. J. Liq. Cryst. Disp..

[47] Chen X.Y., Tian Z. (2017). Recent progress in terahertz dynamic modulation based on graphene. Chin. Opt..

[48] Li T., Zhang M.-L., Wang F., Zhang D.-M., Wang G.-P. (2017). Fabrication of optical waveguide amplifiers based on bonding-type NaYF4: Er nanoparticles-polymer. Chin. Opt..

[49] Yang J.H., Pan M.Q., Shi R.X., Yang L.L., Wang J., Kong X.W., Yang W.Q., Wang D.D., Zhou Z. (2017). Novel Fe_3_O_4_ hollow microspheres: Nontemplate hydrothermal synthesis, superparamagnetism and biocompatibility. Nanosci. Nanotech. Let..

[50] Shah S.T., Yehye W.A., Saad O., Simarani K., Chowdhury Z.Z., Alhadi A.A., Al-Ani L.A. (2017). Surface functionalization of iron oxide nanoparticles with gallic acid as potential antioxidant and antimicrobial agents. Nanomaterials.

[51] Jin C.J., Han J., Chu F.Y., Wang X.X., Guo R. (2017). Fe_3_O_4_@PANI hybrid shell as multifunctional support for au nanocatalysts with remarkably improved catalytic performance. Langmuir.

[52] Miao P., Tang Y.G., Wang L. (2017). DNA modified Fe_3_O_4_@Au magnetic nanoparticles as selective probes for simultaneous detection of heavy metal ions. ACS Appl. Mater. Interfaces.

[53] Baskakov A.O., Solov’eva A.Y., Ioni Y.V., Starchikov S.S., Lyubutin I.S., Khodos I.I., Avilov A.S., Gubin S.P. (2017). Magnetic and interface properties of the core-shell Fe_3_O_4_/Au nanocomposites. Appl. Surf. Sci..

[54] Li X.A., He Y.Y., Sui H., He L. (2018). One-step fabrication of dual responsive lignin coated Fe_3_O_4_ nanoparticles for efficient removal of cationic and anionic dyes. Nanomaterials.

[55] Wang W.T., Tang B.T., Wu S.L., Gao Z.M., Ju B.Z., Teng X.X., Zhang S.F. (2017). Controllable 5-sulfosalicylic acid assisted solvothermal synthesis of monodispersed superparamagnetic Fe_3_O_4_ nanoclusters with tunable size. J. Magn. Magn. Mater..

[56] Ghosh R., Pradhan L., Devi Y.P., Meena S.S., Tewari R., Kumar A., Sharma S., Gajbhiye N.S., Vatsa R.K., Pandey B.N. (2011). Induction heating studies of Fe_3_O_4_ magnetic nanoparticles capped with oleic acid and polyethylene glycol for hyperthermia. J. Mater. Chem..

[57] Ayyappan S., Thoguluva R.R., John P. (2018). Superior thermal stability of polymer capped Fe_3_O_4_ magnetic nanoclusters. J. Am. Ceram. Soc..

[58] Kazeminezhad I., Mosivand S. (2014). Phase Transition of Electrooxidized Fe_3_O_4_ to γ and α-Fe_2_O_3_ Nanoparticles Using Sintering Treatment. Acta Phys. Pol. A.

[59] Zhang M., Xia P., Wang L., Zheng J., Wang Y., Xu J., Wang L. (2014). Synthesis and fabrication of CNTs/Fe_3_O_4_@Pdop@Au nanocables by a facile approach. RSC Adv..

[60] Matioli E., Brinkley S., Kelchner K.M., Hu Y.L., Nakamura S., DenBaars S., Speck J., Weisbuch C. (2012). High-brightness polarized light-emitting diodes. Light Sci. Appl..

[61] Li L., Guo W., Yan Y., Lee S., Wang T. (2013). Label-free super-resolution imaging of adenoviruses by submerged microsphere optical nanoscopy. Light Sci. Appl..

[62] Wang D.D., Wang W.L., Huang M.Y., Lek A., Lam J., Mai Z.H. (2014). Failure mechanism analysis and process improvement on time-dependent dielectric breakdown of Cu/ultra-low-k dielectric based on complementary Raman and FTIR spectroscopy study. AIP Adv..

[63] Xing G., Wang D., Yi J., Yang L., Gao M., He M., Yang J., Ding J., Sum T.C., Wu T. (2010). Correlated d0 ferromagnetism and photoluminescence in undoped ZnO nanowires. Appl. Phys. Lett..

[64] Cheng X., Zhang J., Ding T., Wei Z., Li H., Wang Z. (2013). The effect of an electric field on the thermomechanical damage of nodular defects in dielectric multilayer coatings irradiated by nanosecond laser pulses. Light Sci. Appl..

[65] Wang D.D., Xing G.Z., Yan F., Yan Y.S., Li S. (2014). Ferromagnetic (Mn, N)-codoped ZnO nanopillars array: Experimental and computational insights. Appl. Phys. Lett..

[66] Pincella F., Isozaki K., Miki K. (2014). A visible light-driven plasmonic photocatalyst. Light Sci. Appl..

[67] Xing G.Z., Fang X.S., Zhang Z., Wang D.D., Huang X., Guo J., Liao L., Zheng Z., Xu H.R., Yu T. (2010). Ultrathin single-crystal ZnO nanobelts: Ag-catalyzed growth and field emission property. Nanotechnology.

[68] Wang B., Qu S. (2014). Absorption spectra and near-electric field enhancement effects of Au- and Ag-Fe_3_O_4_ dimers. Appl. Surf. Sci..

[69] Zhang X., Song L., Cai L., Tian X., Zhang Q., Qi X., Zhou W., Zhang N., Yang F., Fan Q. (2015). Optical visualization and polarized light absorption of the single-wall carbon nanotube to verify intrinsic thermal applications. Light Sci. Appl..

[70] Xing G.Z., Yi J.B., Wang D.D., Liao L., Yu T., Shen Z.X., Huan C.H.A., Sum T.C., Ding J., Wu T. (2009). Strong correlation between ferromagnetism and oxygen deficiency in Cr-doped In_2_O_3−δ_ nanostructures. Phys. Rev. B.

[71] Xing G.Z., Yi J.B., Tao J.G., Liu T., Wong L.M., Zhang Z., Li G.P., Wang S.J., Ding J., Sum T.C. (2008). Comparative study of room-temperature ferromagnetism in cu-doped zno nanowires enhanced by structural inhomogeneity. Adv. Mater..

[72] Tran V.T., Zhou H.J., Lee S., Hong S.C., Kim J., Jeong S., Lee J. (2015). Magnetic-assembly mechanism of superparamagnetoplasmonic nanoparticles on a charged surface. ACS Appl. Mater. Interfaces.

[73] Ye M., Wei Z., Hu F., Wang J.X., Ge G.L., Hu Z.Y., Shao M.W., Lee S., Liu J. (2015). Fast assembling microarrays of superparamagnetic Fe_3_O_4_@Au nanoparticle clusters as reproducible substrates for surface-enhanced Raman scattering. Nanoscale.

[74] Du J.J., Xu J.W., Sun Z.L., Jing C.Y. (2016). Au nanoparticles grafted on Fe_3_O_4_ as effective SERS substrates for label-free detection of the 16 EPA priority polycyclic aromatic hydrocarbons. Anal. Chim. Acta.

[75] Chen J., Pang S., He L., Nugen S.R. (2016). Highly sensitive and selective detection of nitrite ions using Fe_3_O_4_@SiO_2_/Au magnetic nanoparticles by surface-enhanced raman spectroscopy. Biosens. Bioelectron..

[76] Li F., Yu Z.F., Zhao L.Y., Xue T. (2016). Synthesis and application of homogeneous Fe_3_O_4_ core/Au shell nanoparticles with strong SERS effect. RSC Adv..

[77] Jiang G.M., Huang Y.X., Zhang S., Zhu H.Y., Wu Z.B., Sun S.H. (2016). Controlled synthesis of Au-Fe heterodimer nanoparticles and their conversion into Au Fe_3_O_4_ heterostructured nanoparticles. Nanoscale.

[78] Freitas M.C.C., Couto M.M., Barroso M.F., Pereira C., Delos-Santos-Álvarez N., Miranda-Ordieres A.J., Lobo-Castañón M.J., Delerue-Matos C. (2016). Highly monodisperse Fe_3_O_4_@Au superparamagnetic nanoparticles as reproducible platform for genosensing genetically modified organisms. ACS Sens..

[79] Abdullaalmamun M., Kusumoto Y., Zannat T., Horieand Y., Manaka H. (2013). Au-ultrathin functionalized core–shell (Fe_3_O_4_@Au) monodispersed nanocubes for a combination of magnetic/plasmonic photothermal cancer cell killing. RSC Adv..

[80] Zhao H.Y., Liu S., He J., Pan C.C., Li H., Zhou Z.Y., Ding Y., Huo D., Hu Y. (2015). Synthesis and application of strawberry-like Fe_3_O_4_-Au nanoparticles as CT-MR dual-modality contrast agents in accurate detection of the progressive liver disease. Biomaterials.

[81] Wang L.Y., Luo J., Fan Q., Suzuki M., Suzuki I.S., Engelhard M.H., Lin Y.H., Kim N., Wang J.Q., Zhong C.J. (2005). Monodispersed Core-Shell Fe_3_O_4_@Au Nanoparticles. J. Phys. Chem. B.

[82] Hong L.R., Zhao J., Lei Y.M., Yuan R., Zhuo Y. (2017). Efficient electrochemiluminescence from Ru(bpy)_3_^2+^ enhanced by three-layer porous Fe_3_O_4_@SnO_2_@Au nanoparticles for label-free and sensitive bioanalysis. Electrochim. Acta.

[83] Wang Y., Li H., Zhang J.J., Yan X.Y., Chen Z.X. (2016). Fe_3_O_4_ and Au nanoparticles dispersed on graphene support as a highly active catalyst toward the reduction of 4-nitrophenol. Phys. Chem. Chem. Phys..

[84] Xia Q.D., Fu S.S., Ren G.J., Chai F., Jiang J.J., Qu F.Y. (2016). Fabrication of Fe_3_O_4_@Au hollow spheres with recyclable and efficient catalytic properties. New J. Chem..

[85] Yu L., Zhang Y.T., Chen R., Zhang D.H., Wei X.H., Chen F., Wang J.X., Xu M.T. (2015). A highly sensitive resonance light scattering probe for Alzheimer’s amyloid-β peptide based on Fe_3_O_4_@Au composites. Talanta.

[86] Hou Y.H., Yuan H.L., Chen H., Shen J.H., Li L.C. (2017). Controlled fabrication and microwave absorbing mechanism of hollow Fe_3_O_4_@C microspheres. Sci. China Chem..

[87] Kwizera E.A., Chaffin E., Wang Y.M., Huang X.H. (2017). Synthesis and properties of magnetic-optical core-shell nanoparticles. RSC Adv..

[88] Mitra A., Mohapatra J., Meena S.S., Tomy C.V., Aslam M. (2012). Verwey Transition in Ultrasmallsized Octahedral Fe_3_O_4_ Nanoparticles. J. Phys. Chem. C.

[89] Zhu Y.M., Zhou X.W., Chen D.S., Li F., Xue T., Farag A.S. (2017). Ternary Fe_3_O_4_@PANI@Au nanocomposites as a magnetic catalyst for degradation of organic dyes. Sci. China Technol. Sci..

[90] Zhang X., Ding S.N. (2017). Sandwich-structured electrogenerated chemiluminescence immunosensor based on dual-stabilizers-capped CdTe quantum dots as signal probes and Fe_3_O_4_-Au nanocomposites as magnetic separable carriers. Sensor. Actuators B Chem..

[91] Hu R., Zheng M.X., Wu J.C., Li C., Shen D.Q., Yang D., Li L., Ge M.F., Chang Z.M., Dong W.F. (2017). Core-shell magnetic gold nanoparticles for magnetic field-enhanced radio-photothermal therapy in cervical cancer. Nanomaterials.

[92] Wang Z.Y., Su R.N., Wang D., Wang J.X., Pu Y., Chen J.F. (2017). Sulfurized graphene as efficient metal-free catalysts for reduction of 4-nitrophenol to 4-aminophenol. Ind. Eng. Chem. Res..

[93] Liu X., Li Y., Xing Z., Zhao X.H., Liu N.N., Chen F.Y. (2017). Monolithic carbon foam-supported Au nanoparticles with excellent catalytic performance in a fixed-bed system. New J. Chem..

