# Supplementary material for: Enhanced Catalytic Reduction of 4-Nitrophenol Driven by Fe3O4-Au Magnetic Nanocomposite Interface Engineering: From Facile Preparation to Recyclable Application
Source: Nanomaterials (Basel). 2018 May 22;8(5):353. doi: 10.3390/nano8050353 (PMC5977367; doi:10.3390/nano8050353)
Supplement: Supplementary file 1 [file nanomaterials-08-00353-s001.pdf]

Enhanced Catalytic Reduction of 4-Nitrophenol Driven by Fe_3O_4-Au Magnetic Nanocomposite Interface Engineering: From Facile Preparation to Recyclable Application

Yue Chen [1,2,†], Yuanyuan Zhang [1,2], Qiangwei Kou [1,2], Yang Liu [1,2,*], Donglai Han [3,†], Dandan Wang [4], Yantao Sun [1,2], Yongjun Zhang [1,2], Yaxin Wang [1,2], Ziyang Lu [5], Lei Chen [1,2], Jinghai Yang [1,2] and Scott Guozhong Xing [6,*]

1 College of Physics, Jilin Normal University, Siping 136000, China; 17649973053@163.com (Y.C.); 13944139606@163.com (Y.Z.); 13944949603@163.com (Q.K.); syt@jlnu.edu.cn (Y.S.); yjzhang@jlnu.edu.cn (Y.Z.); wangyaxin1010@126.com (Y.W.); chenlei@jlnu.edu.cn (L.C.); jhyang1@jlnu.edu.cn (J.Y.)
2 Key Laboratory of Functional Materials Physics and Chemistry of the Ministry of Education, Jilin Normal University, Changchun 130103, China
3 School of Materials Science and Engineering, Changchun University of Science and Technology, Changchun 130022, China; dlhan_1015@cust.edu.cn
4 Technology Development Department, GLOBALFOUNDRIES (Singapore) Pte. Ltd., 60 Woodlands Industrial Park D, Street 2, Singapore 738406, Singapore; DANDAN.WANG@globalfoundries.com
5 School of Environment and Safety Engineering, Jiangsu University, Zhenjiang 212013, China; lzy@mail.ujs.edu.cn
6 United Microelect Corp. Ltd., 3 Pasir Ris Dr 12, Singapore 519528, Singapore
* Correspondence: liuyang@jlnu.edu.cn (Y.L.); Scott_Xing@UMC.com or guozhongupenn@gmail.com (S.G.X);
Tel./Fax: +86-434-329-4566 (Y.L. & S.G.X.)
† The authors have the equal contribution to the manuscript.

Received: 23 April 2018; Accepted: 18 May 2018; Published: date

Abstract: In this work, we report the enhanced catalytic reduction of 4-nitrophenol driven by Fe_3O_4-Au magnetic nanocomposite interface engineering. A facile solvothermal method is employed for Fe_3O_4 hollow microspheres and Fe_3O_4-Au magnetic nanocomposite synthesis via a seed deposition process. Complementary structural, chemical composition and valence state studies validate that the as-obtained samples are formed in a pure magnetite phase. A series of characterizations including conventional scanning/transmission electron microscopy (SEM/TEM), Mössbauer spectroscopy, magnetic testing and elemental mapping is conducted to unveil the structural and physical characteristics of the developed Fe_3O_4-Au magnetic nanocomposites. By adjusting the quantity of Au seeds coating on the polyethyleneimine-dithiocarbamates (PEI-DTC)-modified surfaces of Fe_3O_4 hollow microspheres, the correlation between the amount of Au seeds and the catalytic ability of Fe_3O_4-Au magnetic nanocomposites for 4-nitrophenol (4-NP) is investigated systematically. Importantly, bearing remarkable recyclable features, our developed Fe_3O_4-Au magnetic nanocomposites can be readily separated with a magnet. Such Fe_3O_4-Au magnetic nanocomposites shine the light on highly efficient catalysts for 4-NP reduction at the mass production level.

Keywords: Fe_3O_4 hollow microspheres; Fe_3O_4-Au magnetic nanocomposites; catalytic reduction; 4-nitrophenol

Figure S1

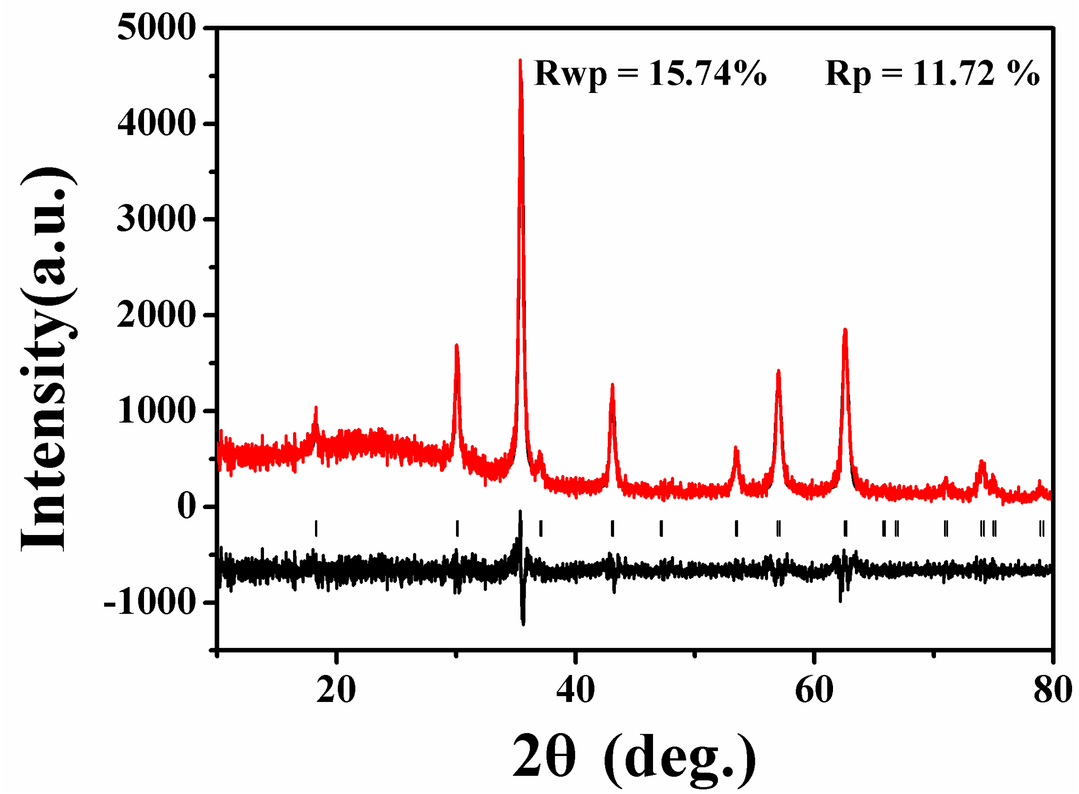

Figure S1. Pawley refinement of the XRD pattern of the pure Fe_3O_4 hollow microspheres. Red dots, blue lines and olive bars represent the experimental data, the calculated date and the peak position of the sample, respectively. The bottom line in black shows the different experimental-calculated data.

Figure S2

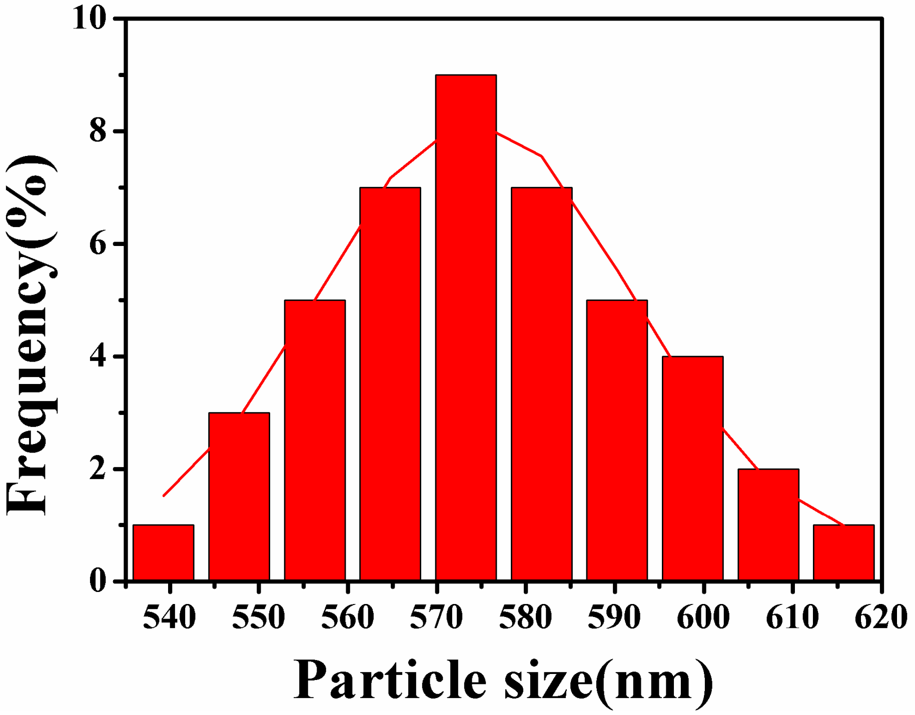

Figure S2. Particle size distribution obtained from the analysis of the TEM images for pure Fe_3O_4 hollow microspheres.

Figure S3

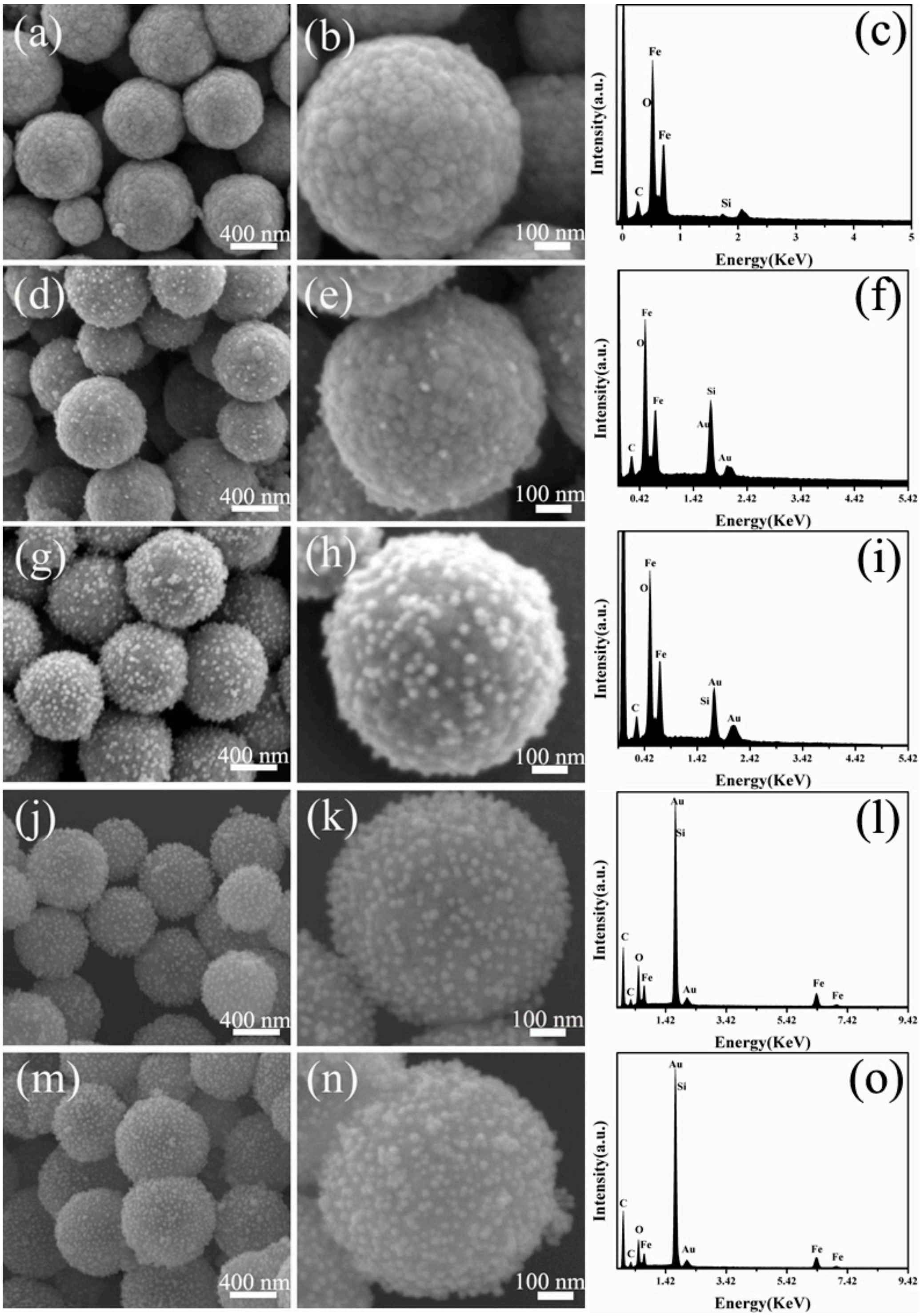

Figure S3. SEM and corresponding EDS images of the as-prepared Fe_3O_4-Au nanocomposites with the different addition quantities of the gold seed colloids (Fe_3O_4 (**a**,**b** and **c**), Fe_3O_4-Au 5 mL (**d**,**e** and **f**), Fe_3O_4-Au 20 mL (**g**,**h** and **i**), Fe_3O_4-Au 40 mL (**j**,**k** and **l**) and Fe_3O_4-Au 60 mL (**m**,**n** and **o**).

Figure S4

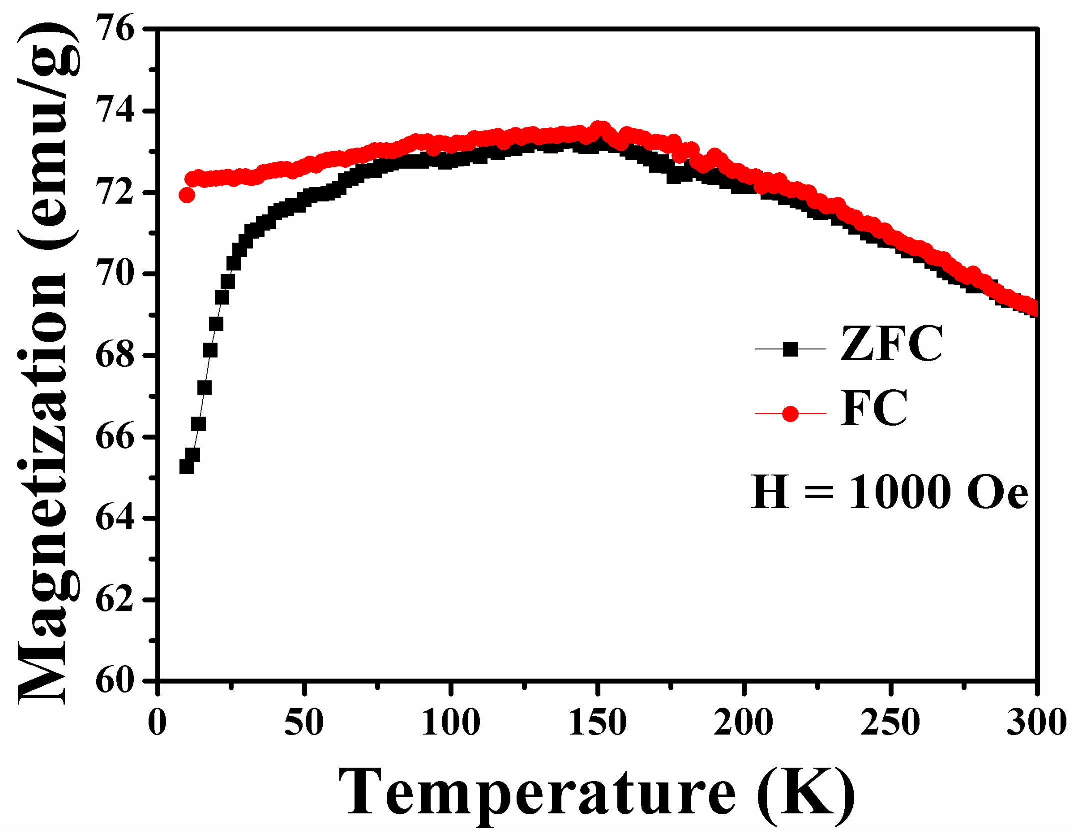

Figure S4. ZFC and FC curves of pure Fe_3O_4 hollow microspheres under an applied field of 1000 Oe.

Figure S5

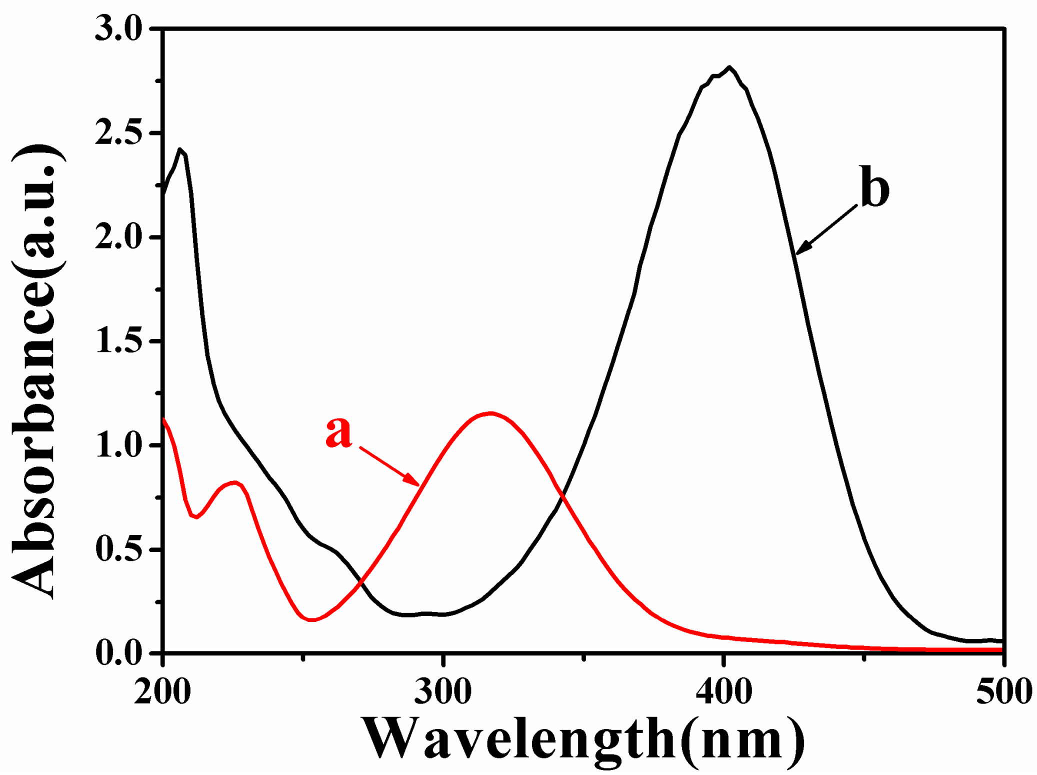

Figure S5. UV-Vis absorption spectra of 4-NP before (red line) and after adding $NaBH_4$ solution (black line).

Figure S6

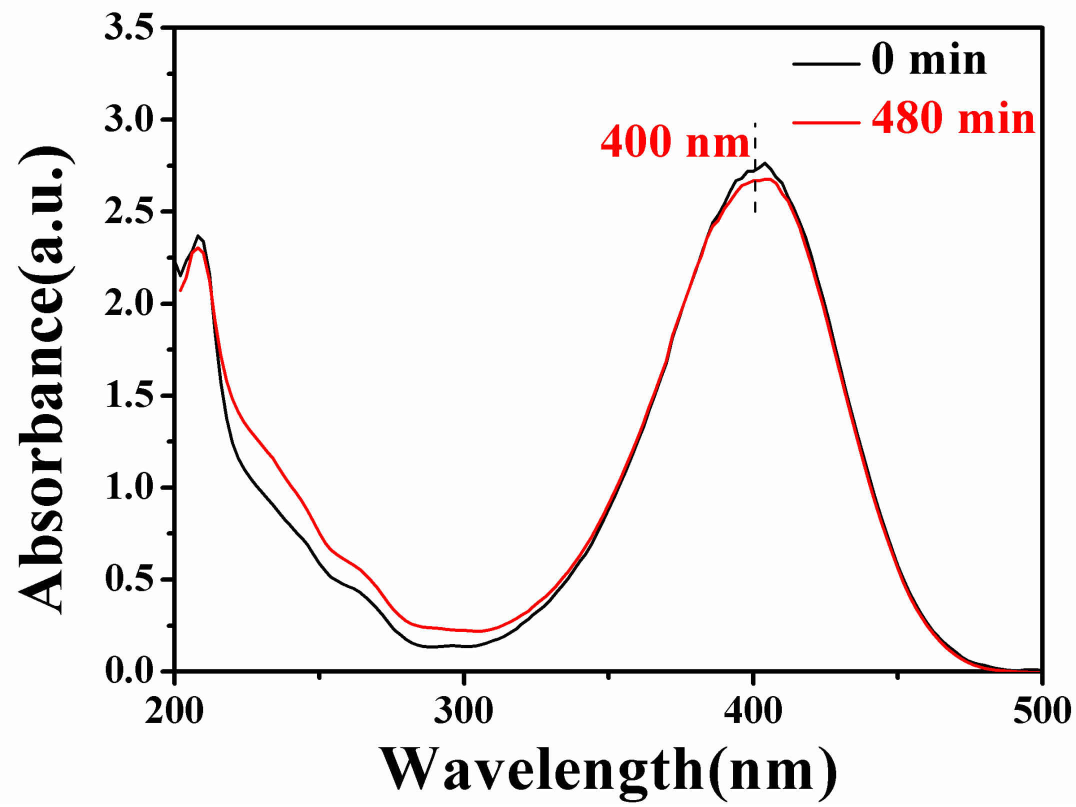

Figure S6. UV-Vis absorption spectra of 4-NP catalyzed by pure Fe_3O_4 hollow microspheres in presence of $NaBH_4$ before and after 480 min.
